# Pain coping skills training for African Americans with osteoarthritis (STAART): study protocol of a randomized controlled trial

**DOI:** 10.1186/s12891-016-1217-2

**Published:** 2016-08-23

**Authors:** Leah A. Schrubbe, Scott G. Ravyts, Bernadette C. Benas, Lisa C. Campbell, Crystal W. Cené, Cynthia J. Coffman, Alexander H. Gunn, Francis J. Keefe, Caroline T. Nagle, Eugene Z. Oddone, Tamara J. Somers, Catherine L. Stanwyck, Shannon S. Taylor, Kelli D. Allen

**Affiliations:** 1 Thurston Arthritis Research Center, University of North Carolina at Chapel Hill, 3300 Thurston Bldg., CB# 7280, Chapel Hill, NC 27599 USA; 2 Department of Medicine, University of North Carolina at Chapel Hill, 125 MacNider Hall CB# 7005, Chapel Hill, NC 27599 USA; 3 Health Services Research and Development Service, Durham VA Medical Center, Durham, NC USA; 4 Department of Medicine, Duke University Medical Center, Durham, NC USA; 5 Department of Biostatistics and Bioinformatics, Duke University Medical Center, Durham, NC USA; 6 Department of Psychiatry and Behavioral Science, Duke University, Durham, NC USA; 7 Department of Psychology, East Carolina University, Greenville, NC USA

**Keywords:** Osteoarthritis, Knee, Hip, Pain coping skills training, Health disparities

## Abstract

**Background:**

African Americans bear a disproportionate burden of osteoarthritis (OA), with higher prevalence rates, more severe pain, and more functional limitations. One key barrier to addressing these disparities has been limited engagement of African Americans in the development and evaluation of behavioral interventions for management of OA. Pain Coping Skills Training (CST) is a cognitive-behavioral intervention with shown efficacy to improve OA-related pain and other outcomes. Emerging data indicate pain CST may be a promising intervention for reducing racial disparities in OA symptom severity. However, there are important gaps in this research, including incorporation of stakeholder perspectives (e.g. cultural appropriateness, strategies for implementation into clinical practice) and testing pain CST specifically among African Americans with OA. This study will evaluate the effectiveness of a culturally enhanced pain CST program among African Americans with OA.

**Methods/Design:**

This is a randomized controlled trial among 248 participants with symptomatic hip or knee OA, with equal allocation to a pain CST group and a wait list (WL) control group. The pain CST program incorporated feedback from patients and other stakeholders and involves 11 weekly telephone-based sessions. Outcomes are assessed at baseline, 12 weeks (primary time point), and 36 weeks (to assess maintenance of treatment effects). The primary outcome is the Western Ontario and McMaster Universities Osteoarthritis Index, and secondary outcomes include self-efficacy, pain coping, pain interference, quality of life, depressive symptoms, and global assessment of change. Linear mixed models will be used to compare the pain CST group to the WL control group and explore whether participant characteristics are associated with differential improvement in the pain CST program. This research is in compliance with the Helsinki Declaration and was approved by the Institutional Review Boards of the University of North Carolina at Chapel Hill, Durham Veterans Affairs Medical Center, East Carolina University, and Duke University Health System.

**Discussion:**

This culturally enhanced pain CST program could have a substantial impact on outcomes for African Americans with OA and may be a key strategy in the reduction of racial health disparities.

**Trial registration:**

ClinicalTrials.gov, NCT02560922, registered 9/22/2015.

## Background

Osteoarthritis (OA) is a leading cause of pain and disability and one of the most commonly diagnosed diseases in the U.S.; about 27 million adults have symptomatic OA [1]. The prevalence of OA is expected to double over the next several decades [2]. In addition to pain and disability, OA has detrimental effects on depression, anxiety, sleep, fatigue, physical activity, weight gain, work participation, and quality of life [3–8]. The rising prevalence of OA and its significant effects on numerous health outcomes highlight the need for effective intervention strategies.

African Americans bear a disproportionate burden of osteoarthritis (OA). A number of studies have shown that African Americans not only experience higher prevalence rates of OA than Caucasians, but also more severe pain, functional limitations, and other adverse outcomes [9–13]. Despite many years of research highlighting the disproportionate burden of OA and other pain-related conditions among African Americans, very little has been done to address these disparities [14]. One review noted that key barriers to moving this research forward have included limited engagement by minority patient groups and a lack of testing of pain management interventions in these groups, including African Americans [15].

Pain Coping Skills Training (CST) is a cognitive-behavioral intervention with shown efficacy to improve OA-related pain and other outcomes [16–22]. However, there has been limited work to obtain perspectives on cultural appropriateness of pain CST among African Americans with OA. Prior research suggests that pain CST may be a promising strategy to address racial disparities in OA-related pain. When compared with Caucasians, African Americans with OA and other chronic pain conditions report greater levels of pain catastrophizing [23, 24], lower perceived ability to cope with and control pain [25, 26], and greater maladaptive coping strategies [13, 25, 27, 28]. These coping patterns can be modified through pain CST [18, 19, 29–31]. In addition, prior studies indicate that pain coping and other psychological factors are key contributors to racial differences in OA-related pain [12, 13]. Based on this promising research, there is a need for a stronger evidence base for the effectiveness of pain CST among African Americans with OA.

This manuscript describes the protocol for a randomized controlled trial examining the effectiveness of a culturally enhanced, telephone-based pain CST program among African Americans with hip or knee OA. The first objective of this project was to “Engage African American patients with OA, their support partners, health care providers, clinic administrators, and public health representatives in a process of evaluating and refining a pain CST program for culturally appropriate content and dissemination potential.” The processes and results for this objective are described in the “Pain CST Program Development” section below. The two aims of the clinical trial are:

### Aim #1

Examine the effectiveness of an 11-session, culturally enhanced, telephone-based pain CST program among African Americans with hip or knee OA.

*We hypothesize that African Americans with symptomatic OA who receive a pain CST intervention will have clinically relevant improvements in pain (Western Ontario and McMasters Universities Osteoarthritis Index; WOMAC) and secondary related outcomes at 12 week follow-up (H*_*1*_*) and 36 week follow-up (H*_*2*_*), compared with a Waiting List (WL) Control group.*

### Aim #2

Examine whether individual patient characteristics (particularly baseline pain catastrophizing score, comorbidity, and duration of OA symptoms) are associated with differential improvement in the pain CST program.

*This aim will involve exploratory analyses of differential treatment effects according to participant characteristics.*

## Methods

This study was reviewed and approved by the Institutional Review Boards of the University of North Carolina at Chapel Hill, Duke University Medical Center, Durham Veterans Affairs Medical Center (VAMC), and East Carolina University (all located in the United States). The funding agency, Patient-Centered Outcomes Research Institute (PCORI), did not have a role in study design and will not have a role in the collection, management, analysis, or interpretation of data.

### Study design

**Fig. 1:**
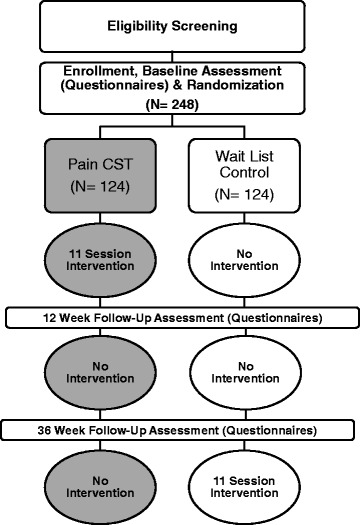
Overview of Trial Design

The Pain Coping **S**kills **T**raining for **A**frican Americans with Osteo**ART**thritis (STAART) study is a parallel-group design, randomized controlled trial with a planned sample size of 248 African American participants with equal allocation to a pain CST group and a (WL) control group (Fig. 1, Overview of Trial Design). Participants are patients with hip or knee OA at the University of North Carolina (UNC) Health Care System or the Durham VAMC (*n* = 124 at each site). Randomization is stratified according to enrollment site and gender to ensure that the groups are balanced in these respects. The three measurement time points are baseline, 12 week follow-up (primary outcome assessment), and 36 week follow-up (maintenance assessment). Following completion of the 36 week assessments, participants assigned to the WL control group will be invited to take part in the CST program. Participants in both study groups will continue with their usual medical care for OA during the full study period.

### Participant eligibility criteria

Participants must meet the following criteria for at least one hip or knee:

- Diagnosis of Hip or Knee OA. This will be identified from UNC Health Care and Durham VAMC electronic medical records or based on participant self-report at screening.
- Current Joint Symptoms. Participants must indicate having pain, aching, stiffness, or swelling in or around their hip or knee with OA on most days for the past month.

Exclusion criteria are shown in Table 1.

**Table 1:** Exclusion criteria

Diagnosis of gout (in knee or hip), rheumatoid arthritis, fibromyalgia, or other systemic rheumatic disease	
Dementia or other memory loss condition	
Active diagnosis of psychosis, serious personality disorder, or current uncontrolled substance abuse disorder	
Total hip/knee replacement surgery, other hip/knee surgery, ACL tear, or other significant knee/hip injury in the past 6 months	
Scheduled for or on a waiting list for joint replacement surgery	
Severely impaired hearing or speech (patients must be able to participate in telephone sessions)	
Unable to speak English	
Participating in another OA intervention or CST study	
Unwilling to be randomized to either study arm	
Lower extremity paralysis	

### Recruitment, enrollment, and randomization

Three general methods of recruitment are being used. The first involves posting of advertisements at study sites and the surrounding communities, inviting patients to self-refer to the study. Second, health care providers at study sites are able to refer patients to the study team directly, with patients’ permission. Providers may also give study brochures to participants. Third, potentially eligible patients are being actively identified from UNC and Durham VAMC medical records, based on OA diagnosis codes; these patients are mailed letters inviting participation, followed by a telephone call. All potential participants are screened for eligibility criteria via telephone. Individuals who meet eligibility criteria and are interested in participating are asked to meet with a study team member to complete consent, HIPAA authorization, and baseline assessments. We are using an enhanced informed consent process that includes education about the research process, participant bill of rights, and perspectives from African Americans who have participated in research. Specifically, participants are mailed the “You’ve Got the Power!” booklet prior to their enrollment visit and shown the “What You Should Know About Clinical Trials” video at the beginning of the enrollment visit. Both of these materials were developed and are distributed by the National Medical Association as a part of Project IMPACT – Increase Minority Participation and Awareness of Clinical Trials [32]. Following baseline assessments, participants are given their randomization assignment over the telephone by a study team member not blinded to group assignment. Participants randomized to the WL group are informed that they will begin the CST program after their follow-up assessments are complete. Randomization is a stratified block randomization with block sizes <10 maintained by the project statistician. The randomization scheme is contained in the study database and study team members who are blinded to treatment group do not have access to this section in the database.

Participants will be withdrawn from the study if they develop any new health problems or other events that would either make participation in the study intervention or measures unsafe or confound study outcomes. These largely mirror study exclusion criteria.

### Pain CST program

#### Pain CST program development

The pain CST program was based on prior clinical trials among patients with various chronic pain conditions [18–20, 22]. In addition, previous work with the CST program incorporated perspectives of African American men with prostate cancer [33]. Building on that work, the CST program was refined in a small, single-arm pilot study among African American Veterans with OA. Then as part of this project, we worked with a broader group of stakeholders to further enhance the program with attention to issues of cultural relevance and future dissemination potential. These stakeholders included African Americans with OA, public health representatives in the field of arthritis, patient advocates, and health care providers. During the course of proposal development, we met with patients and other stakeholders who reviewed several aspects of the project, including the intervention content and dissemination plans. In the first phase of this project, we presented the CST program modules to a group of African American patients with OA, via telephone, and incorporated their input into the program. Following presentation of each module, we asked these stakeholders a series of questions regarding aspects of the program they liked, components or ideas that were difficult to understand, ways in which each skill related or did not relate to their cultural, spiritual, religious or other values, and barriers they may face to incorporating each skill into their normal daily lives. We also sought their input on the intervention handbook. Other members of the study stakeholder panel also provided input on the CST program and handbook. We describe below the enhancements that have been made to enrich cultural sensitivity for each topic and skill as a result of previous related work [33] and the early work on this project.

#### Overview of the pain CST program

The CST program involves 11 weekly sessions, approximately 30–45 min each, delivered via telephone by a trained counselor. Participants are provided with handouts to facilitate each session, along with an audio recording to guide progressive muscle relaxation. A counselor provides instruction in cognitive and behavioral pain coping strategies and also leads participants in guided rehearsals of these skills. Specific skills are described below. Participants are asked to engage in home-based practice of the skills to enhance their application in pain-related situations. During each session, the counselor reviews participants’ home practice, including successes and barriers, encourages problem solving, and works to set goals for application of skills.

#### CST session topics and skills

##### Session 1 – Introduction, Rationale for Pain Coping Skills, Progressive Muscle Relaxation

**Introduction.** Participants are provided with a brief overview of the session formats, as well as the CST participant manual. The importance of regular practice of the coping skills is emphasized. Feedback from patient stakeholders contributed to a decision to frame the CST program as an educational intervention instead of “treatment” or “therapy.”

**Education in the Rationale for CST.** Participants are provided with a brief rationale for the CST intervention, including components of gate control theory. This helps participants to understand how their thoughts, feelings, and behaviors can influence pain, as well as how coping skills can enhance their ability to control pain.

**Progressive Muscle Relaxation.** Participants are also introduced to progressive muscle relaxation in this session. This technique enhances one’s ability to identify and decrease tension throughout the body. This skill involves tensing and relaxing different muscle groups, from the feet to the forehead, while also maintaining deep relaxed breathing. After a description of the technique and a review of its benefits, the CST counselor guides participants through a 15 min relaxation session.

##### Session 2 – Mini Relaxation Practices and Communicating with Significant Others about Pain and Coping

**Mini-Relaxation Practices.** Mini-relaxation is a form of applied relaxation that provides a means to decrease tension and pain when it is not feasible to do a full progressive muscle relaxation session. During this session, the CST counselor guides participants through a mini-relaxation practice that lasts about 30 s. Participants are instructed to take a long breath in, exhale slowly while saying the word “relax” to themselves, and concentrate on the sensations of relaxation flowing throughout the body. Participants are then coached on strategies for applying mini-relaxation practices using both internal cues (e.g., increase in mood or tension) and external cues (e.g., before eating a meal). They are also taught how to use mini-relaxation exercise during activities of daily living that are potentially painful (e.g. prolonged standing, transferring from sitting to standing, and stair climbing).

**Communication with Significant Others about Pain and Coping.** One enhancement we made to the CST program, based on input from our stakeholders and previous work [19, 33], is increased attention to the role of significant others (e.g. spouse, family members, co-workers) in dealing with chronic pain. Specifically, we added a module that provides guidance in speaking and listening strategies, as well as expressive versus decision-making conversations. These are applied to communicating with significant others about pain. Following discussion of these topics, the counselor leads participants in a process of planning effective expressive and decision-making conversations, using examples of relevance to them.

##### Sessions 3 and 4 – Managing Unhelpful Mood

Cognitive restructuring (described as “managing unhelpful mood” for participants in this program), involves teaching participants to recognize relationships between thoughts, feelings, and behaviors [34]. Participants are first taught to identify overly negative, maladaptive thoughts regarding pain and then to replace those with alternative, more realistic and helpful coping thoughts that enhance pain control. Cultural enhancements for this skill have involved regular encouragement for participants to incorporate spiritual or other cultural values into thoughts about pain and pain management.

##### Session 5 – Activity Pacing

In this session, participants are first taught how to recognize the over-activity cycle, which is a common approach to dealing with pain. In this cycle, individuals tend to overdo an activity, require an extended period of rest or recovery, then overdo the activity again or choose to avoid the activity altogether. The over-activity cycle can lead to negative consequences such as increased pain, avoidance of activity, and increased tension. As an alternative to this maladaptive cycle, participants are taught an activity-rest cycling strategy [35]. Participants are asked to identify activities in which they tend to overexert themselves, develop a plan for breaking up those activities into periods of activity and rest (i.e., 20 min of housework followed by 10 min of rest), and gradually increase their activity level and decrease resting periods. One cultural enhancement for this skill has involved emphasizing the role of rest in completing tasks, e.g., taking breaks actually can help individuals to accomplish tasks more easily and effectively. This enhancement was in response to findings of Campbell and colleagues [33] (and confirmed among our patient stakeholders) that particularly among African American men, task completion and contributing to the larger community is a common value. In addition, in this session, participants are encouraged to use their communication skills (Session 2) to explain to others why activity pacing will help them accomplish tasks.

##### Session 6 – Pleasant Activities

Chronic pain can lead to social withdrawal and lowered activity levels. To address this issue, this session teaches participants strategies for remaining engaged in valued activities. The counselor asks participants to brainstorm activities they enjoy or that give them a sense of accomplishment. Participants are also given a list of example pleasant activities based on activities participants have focused on in prior studies. Participants are asked to set weekly goals for scheduling pleasant activities they choose, with a focus on increasing the level and range of these activities over time. Time is devoted to helping participants develop specific plans for integrating appropriate pleasant activities into their daily routine, despite time constraints such as multiple caregiving roles.

##### Session 7 – Pleasant Imagery and Other Distraction Techniques

Participants are trained in four attention diversion methods that can enhance pain control: pleasant imagery, focal point, auditory stimuli, and challenging mental tasks. Pleasant imagery involves shifting attention from an unpleasant situation (e.g., increase in pain) to a pleasant and relaxing scene; this can be used as an adjunct to relaxation [36]. Focal point distraction involves intentionally focusing on a point or object in the environment for 1–2 min. Similarly, auditory stimuli distraction involves focusing on a noise in the environment for 1–2 min. Challenging mental tasks may include counting backwards in increments of any number or thinking of a type of food that starts with every letter in the alphabet.

##### Session 8 – Physical Activity and Osteoarthritis; Session 9 – Weight Management and Osteoarthritis

The CST counselor works with participants to place pain CST in the broader context of OA management. Physical activity and weight management are particularly important lifestyle strategies for managing OA [37, 38]. Although pain CST is the focus of this intervention, we also support participants’ engagement in these other lifestyle approaches, using low literacy materials and intervention scripts we developed in other studies [39]. These materials take a behavioral, goal-setting approach to weight management and physical activity. This order was selected strategically, since each of the pain coping skills will have been introduced by session 8; this allows the counselor to work with participants to integrate those skills in their efforts to change physical activity and dietary patterns. For example, activity-rest cycling is important for helping patients to improve overall physical activity without a significant pain exacerbation. Cognitive restructuring is also important for helping patients to address unhelpful thoughts that link pain to these other behaviors. For example, some individuals respond to pain with unhealthy eating patterns; uncovering and addressing unhelpful pain-related thoughts is a key strategy in changing these patterns.

##### Session 10 – Skills Review and Problem Solving

Different coping skills may be particularly helpful for specific challenging pain-related problem situations (e.g. an unexpected weekend visit from relatives or having to take a long plane trip that involves extensive and prolonged sitting). To facilitate patients’ ability to deal with these situations, the CST counselor reviews all of the skills taught in the program and then teaches participants a 3-step problem solving technique. This technique involves 1) describing the situation, 2) identifying likely difficulties associated with the situation, and 3) identifying a skill or combination of skills that may be best suited for the context.

##### Session 11 – Relapse Prevention and Maintenance

**Relapse Prevention.** Consistent and continued practice of pain coping skills is an integral part of effective pain control. Therefore participants are introduced to a five-step process to help prevent and manage potential setbacks. This involves 1) identifying warning signs of a setback, 2) noticing immediate thoughts and actions during a setback, 3) utilizing a coping skill, 4) reviewing the situation that lead to a setback, and 5) making an immediate plan for dealing with setbacks.

**Maintenance.** In order to encourage continued practice of pain coping skills, the CST counselor reviews each skill and their unique benefits. Finally, the CST counselor helps participants develop an individualized home program that includes a daily practice schedule and short- and long-term goals.

#### CST counselor training and adherence

The counselor received training in the pain CST protocol, including role play sessions, by experienced co-investigators (TS, LC). Counselor training also included issues related to cultural sensitivity, by a co-investigator with expertise in this area (LC). Following initial training, three strategies are being used to ensure adherence to the pain CST program protocol: 1) use of detailed scripts for each module, 2) regular supervision sessions with co-investigators experienced with the pain CST program (TS, FJK, LC), and 3) audio-recordings of a subset of intervention calls which are reviewed by experienced co-investigators and rated for adherence.

### Measures

All study assessments are conducted by trained research assistants blinded to the participants’ randomization assignment. The baseline and 12 week assessments are conducted in person and the 36 week assessment is conducted via telephone. To facilitate participant retention and completion of follow up, there is some allowance for the 12 week assessment to be conducted via telephone in cases when participants are unable to return to the study site. Participants are paid $50 for completion of the baseline assessment, $50 for the 12 week assessment, and $25 for the 36 week assessment.

### Primary outcome

#### Western Ontario and McMasters Universities Osteoarthritis Index (WOMAC) pain subscale

The WOMAC pain subscale is one of the most commonly used measures of pain among patients with lower extremity OA. It includes 5 items rated on a Likert scale of 0 (no symptoms) to 4 (extreme symptoms). The reliability and validity of the WOMAC total score and subscales have been confirmed [40], and this scale has been widely used in trials of behavioral interventions for patients with hip and knee OA, confirming its sensitivity to change in these types of interventions.

### Secondary outcomes

#### WOMAC total score and function subscale

In addition to the pain subscale, the WOMAC includes stiffness (two items) and function (17 items) subscales. The WOMAC total score and function subscale are also common patient-centered outcomes for patients with lower extremity OA. We are assessing the function subscale separately because of its importance as an outcome among patients with OA.

#### Depressive symptoms – patient health questionnaire-8 (PHQ-8)

The PHQ-8 is an 8-item survey derived from the Primary Care Evaluation of Mental Disorders (PRIME-MD) diagnostic tool, and consists of items corresponding to the depression criteria listed in the *Diagnostic and Statistics Manual Fourth Edition* (DSM-IV) [41]. Each of the eight questions is scored as 0 (not at all) to 3 (nearly every day), so that total scores range from 0 to 24.

#### Patient global impression of change

This scale evaluates participants’ perspectives on overall changes in their joint pain during the study period. This single-item measure asks participants to describe their change in pain on a 7-point rating scale with the following options: “very much improved,” “much improved,” “minimally improved,” “no change,” “minimally worse,” “much worse,” and “very much worse.”

#### Coping Strategies Questionnaire (CSQ)

This scale includes 48 items that assess 6 cognitive domains (Catastrophizing, Diverting Attention, Ignoring Sensations, Coping Self-Statements, Reinterpreting Pain Sensations, Praying-Hoping) and one behavioral domain (Increasing Behavioral Activities). Each domain includes six items, and participants rate the frequency of their use of specific coping strategies on a 7-point Likert scale from 0 (“Never do that”) to 6 (“Always do that”). The scale also includes two items that assess participants’ perceptions of the effectiveness of their pain coping skills, i.e. their subjective ability to control or decrease their pain, using a similar 7-point Likert scale. The CSQ is the most commonly used measure of coping among individuals with chronic pain, and its measurement properties have been confirmed in patients with a variety of pain-related conditions [42, 43].

#### Arthritis self-efficacy scale

This scale includes 8 items asking respondents how certain they are that they can perform specific activities or tasks. Items are scored on a Likert Scale (1 = very uncertain to 10 = very certain). This scale has shown acceptable construct validity (by its significant associations with pain, disability, and depression), internal reliability (alpha = 0.76–0.89), and test-retest reliability (Pearson *r* = 0.71–0.85). Higher scores on this scale have been significantly associated with improved health outcomes and protection against poor functional outcomes [44, 45].

#### Health-Related Quality of Life (HRQoL)

The Short-Form 12 Health Survey (SF-12) is a 12-item validated measure that covers domains of general health, physical health, work and activity limitations, and emotional health [46]. We are including a measure of HRQoL based on feedback from our patient stakeholders, who stressed that OA affects many aspects of life and recommended that we broadly assess quality of life in this study.

#### PROMIS pain interference instrument (Short Form)

The PROMIS Pain Interference (Short Form 6a) instrument measures the self-reported consequences of pain across aspects of life including social, cognitive, emotional, physical, and recreational activities; this instrument refers to the past 7 days [47]. This validated scale has five response options, with scores ranging from 1 to 5.

#### Pain medication use

Participants are asked to bring to their study visits (or bring to the telephone) all medications (prescription and non-prescription) they are currently taking for their arthritis symptoms. For each medication, the study team records the medication name, medication class, and frequency of taking the medication. We also use a single-item measure that asks participants whether their overall pain medication use for OA has increased, decreased, or stayed about the same since the beginning of the study.

#### Arthritis self-efficacy for pain communication scale – patient version

This 7-item instrument assesses patients’ level of confidence in communicating their pain to their partner and receiving understanding and a helpful response from their partner [48]. Items are rated on a scale from 10 (“very uncertain”) to 100 (“very certain”).

#### Starting the conversation: diet (STC)

The STC is an 8-item food frequency instrument that evaluates dietary assessment and intervention in a clinical setting [49]. Response options for the survey are organized into three columns; one column indicating the most healthful dietary practices (scored 0), the 2^nd^ column indicating less healthful practices (scored 1), and the 3^rd^ column indicating the least healthful practices (scored 2).

#### Brief fear of movement scale

The Brief Fear of Movement Scale is a 6-item scale for assessing fear of movement in OA [50]. The scale specifically assesses activity avoidance due to pain-related fear of movement. All items are measured on a 4-point scale from “strongly agree” to “strongly disagree.”

#### Pain Catastrophizing Scale (PCS)

This 13-item instrument asks participants to reflect on past painful experiences and to indicate the degree to which they experienced each of the thoughts or feelings when experiencing pain. The PCS includes three subscales – rumination, magnification, and helplessness. The PCS is a widely used measure of catastrophic thinking related to pain [51].

#### Time missed from work

This is a single item measure asking participants how many work hours they have missed in the past month due to their OA pain and other symptoms and related healthcare visits.

### Demographic and clinical characteristics

Participant characteristics include age, race/ethnicity, gender, marital status, household financial state (with low income defined as self-report of “just meeting basic expenses” or “don’t even have enough to meet basic expenses”), education level, work status, religiosity (Duke University Religion Index [52]), body mass index, physical activity (Yale Physical Activity Survey [53]), tobacco and alcohol use, duration of OA symptoms, general self-rated health, comorbid illnesses (Self-Administered Comorbidity Questionnaire [54]), and baseline pain catastrophizing (CSQ subscale [42, 43]).

### Participant feedback on CST intervention

At the 12 week follow up assessment, following completion of the CST intervention, participants in the CST group will be asked a series of feedback questions related to specific content (e.g., perceived usefulness and suggestions related to each coping skill) and process (e.g., the number and duration of sessions) of the program. This information will be used by the study team, in conjunction with the stakeholder panel, to refine the program prior to dissemination of deliverables.

### Data analyses

The primary and secondary analyses will be conducted on an intent-to-treat basis. Participants will be analyzed in the arm to which they were randomized, regardless of intervention adherence, using all follow-up data. Additional supporting analyses focusing on alternative, more restrictive analytic cohorts (e.g., as treated) will be considered as exploratory analyses to provide additional information about the impact of magnitude of exposure to the intervention.

### Analysis of specific aim #1

We will use a linear mixed model (LMM) that will account for the correlation between a participant’s repeated outcome measurements over time. Because of the small number of time points, we will apply an unstructured covariance matrix to take into account the within-patient correlation between repeated measures. We will estimate the parameters in the model using the SAS procedure MIXED (Cary, NC), and will test appropriate parameters for a difference in mean WOMAC pain scores between the CST and WL groups at specified time points. A constrained longitudinal data model (cLDA) will be fit, in which baseline WOMAC pain is modeled as a dependent variable in conjunction with the constraint of a common baseline mean across the treatment arms [55]. The cLDA model is comparable to an ANCOVA model, equivalent when there is no missing data. However, unlike an ANCOVA, subjects who are missing follow-up measurements are included in the model because baseline is part of the response vector. For improvement in precision, the model will also be adjusted for stratification variables of enrollment site and gender [56]. Similar procedures will be used for all continuous secondary outcomes. The secondary outcomes for pain medication use are either dichotomous or count type variables. We will fit a generalized logit model [57] using the SAS procedure NLMIXED for these outcomes.

### Analysis of specific aim #2

Patients may vary in their response to the CST program; this variation is known as heterogeneity of treatment effects (HTE). We have selected three a priori patient characteristics (baseline pain catastrophizing score, comorbidity, and duration of OA symptoms) and will conduct a separate descriptive HTE for each. Our general steps in this secondary analysis will be to add the patient characteristic main effect, as well as the interaction variables, to the linear mixed model defined above for primary analysis. We will examine the parameter estimates and 95 % CI’s of appropriate parameters to determine whether there is evidence of HTE. New state-of-the art modeling methods have taken the exploration of HTE to the next level, making it possible to explore and identify multidimensional subgroups exhibiting heterogeneous treatment effects. We will explore whether the a priori defined patient characteristics define multidimensional subgroups that exhibit HTE. We will utilize two different analytic strategies for doing so: multivariable logistic regression (LR) [58, 59] and recursive partitioning (RP) [60–62]. Our general steps in this secondary analysis will be: construction of outcome variables; identification of multidimensional subgroups via LR and RP; and, finally, examination of treatment effects within the multidimensional subgroups.

### Missing data

Our plans for preventing and dealing with missing data follow the guidelines set forth by the National Research Council’s Panel on Handling Missing Data in Clinical Trials. Our goal is to achieve less than 20 % attrition, which is very reasonable based on our prior and ongoing OA studies that include large proportions of African Americans [39, 63, 64]; we will use the same strategies of reminder calls, reminder letters and flexible scheduling to minimize attrition. Our main analysis technique for the primary outcomes, general linear mixed models via maximum likelihood estimation, implicitly accommodates missingness when missingness is due either to treatment, to prior outcome, or to other baseline covariates included in the model, defined as missing at random [57]. Therefore, inferences will be valid even if we have differential dropout by intervention arm. However, as a first sensitivity analysis, we will construct a general, multivariate imputation model using all observed pain measurements, treatment arm, and any covariates predictive of missingness [65]. If the probability of dropout is related to the actual missing response (which is unobserved because it is missing) or to other unobserved quantities, the missing data due to dropout is considered missing not at random (MNAR) or nonignorable [66]. We propose as a second sensitivity analysis to explore MNAR methods, including pattern-mixture models and the toolkit of methods as presented in O’Kelly and Ratitch [67].

### Sample size

The sample size estimate of *n* = 124 per arm was based on the primary research question and the 36-week follow-up time as that is the most conservative test due to higher attrition at the 36 week time point versus 12 weeks. Sample size calculations used methods appropriate for ANCOVA type analyses [68], which are equivalent in terms of efficiency to our proposed linear models in randomized trials [55]. Based on previous data, we assumed a correlation of 0.6 between baseline and follow-up WOMAC pain scores, and an SD of 3.9. With 80 % power, alpha = 0.05, SD = 3.9, rho = 0.60, and a conservative 20 % attrition rate by 36 weeks, we need to enroll 124 patients per group to detect a 1.3 point difference in mean WOMAC pain scores at 36 weeks between the CST group and the WL control group. Based on a mean baseline WOMAC pain score of 9.2, this corresponds to approximately a 14 % improvement or 0.33 (medium) effect size difference in WOMAC pain, which is a clinically relevant improvement [69]. Similarly, for the remaining secondary outcomes, we are powered to detect a 0.33 effect size difference for the CST arm compared to the WL control group. We have greater than 80 % power to detect a 1.3 point difference in pain scores between CST and WL at 12 weeks.

### Data management and monitoring

Every effort will be made to protect the confidentiality of participant data. Study data are kept on a secure UNC computer server that only approved study personnel have access to. Study tracking data are entered into a secure, password-protected web-based database we have developed at UNC. Screening and outcome measures are integrated into this database and are entered in real time during study assessments. This database provides customized reports that have important functions for monitoring a clinical trial. These reports provide updates on the numbers of participants enrolled, excluded, withdrew, etc. These reports also facilitate easy checking of screening and outcomes data so the team can monitor for unexpected amounts of missing data. Study team members can generate regular reports, based on a CONSORT diagram that describes all of these metrics related to study flow, as well as participant refusal, ineligibility, and withdrawal reasons.

Because this is a study with minimal risk, data monitoring is performed by the Principal Investigator and Institutional Review Board (IRB). This study does not report to a data monitoring committee. All adverse events are reported to and reviewed by the Principal Investigator. Any unanticipated problems involving risks to subjects or others are reported to the IRBs per required timelines. Any important protocol modifications are reported to the UNC and Durham VAMC IRBs as well as the funding agency, PCORI.

### Data access and dissemination

Study contractual agreements encourage openness in research and making research data available for purposes of replication and reproducibility. As such, the researchers intend to provide access to project data in a manner that is consistent with applicable privacy, confidentiality, and other legal requirements. Final results of the research project will be submitted to ClinicalTrials.gov as well as the funding agency, PCORI, to ensure that research findings are made available to clinicians, patients, and the general public. The researchers plan to publish and present study results, as available, and dissemination will be conducted to relevant parties via our stakeholder panel.

For all published manuscripts of this project, we are following the authorship guidelines set forth by the International Committee of Medical Journal Editors (ICMJE). All authors will meet this criteria and anyone who meets this criteria will be given authorship. Those who have contributed to the paper but do not meet the specific criteria set forth by the ICMJE will be included in the acknowledgements.

## Discussion

This study has several important features. First, to our knowledge, it is the first randomized clinical trial evaluating the effectiveness of a culturally enhanced, telephone-based pain coping skills training program for African Americans with knee or hip OA. Pilot study results suggest that our pain CST program, with cultural tailoring, may be particularly beneficial for African Americans with OA, but this larger clinical trial is needed to rigorously examine its effectiveness. Second, this study takes a pragmatic approach, including participants who experience the spectrum of OA symptom severity and incorporating minimal exclusion criteria. This is important for generalizability of the findings to real-world settings and a large variety of patients. Third, we are collaborating with a diverse stakeholder panel, comprised of African American patients with knee or hip OA, clinicians, community partners and organizations that seek to improve health outcomes among African Americans and other racial/ethnic minority groups, and representatives of national organizations seeking to improve outcomes for people with OA. This group has made key contributions to the development of the pain CST program and study design and will continue to provide feedback on the intervention and ongoing clinical trial.

We recognize there are limitations to this study. For ethical reasons, study participants in each group are permitted to receive usual OA treatments during the study period, and this could impact pain and other outcomes. However, we are purposefully enrolling patients who are currently under care for OA in one of two health care systems. Therefore, we are examining whether the pain CST program results in improvements in outcomes beyond usual OA care. For logistical reasons, we are not obtaining radiographs from study participants at baseline, and we therefore cannot characterize participants with respect to radiographic stage of OA. However, we are selecting participants on the basis of a diagnosis of OA in the medical record, along with self-report of physician diagnosis of OA.

In summary, results of this study will provide important information regarding the potential for pain CST programs to reduce racial disparities in OA-related pain. If this culturally enhanced CST program is confirmed to result in clinically meaningful changes among African Americans with OA, incorporation into clinical settings could have a substantial impact. This is particularly important given the high rates of OA and greater symptom burden among African Americans who suffer from this condition.

## Abbreviations

ANCOVA: Analysis of covariance
CSQ: Coping strategies questionnaire
CST: Coping skills training
DSM-IV: Diagnostic and statistics manual fourth edition
GLMM: General linear mixed model
HRQoL: Health-related quality of life
HTE: Heterogeneity of treatment effects
ICMJE: International Committee of Medical Journal Editors
IRB: Institutional review board
OA: Osteoarthritis
PCS: Pain catastrophizing scale
PRIME-MD: Primary care evaluation of mental disorders
SD: Standard deviation
STC: Starting the conversation: diet
UNC: University of North Carolina at Chapel Hill
VAMC: Veterans affairs medical center
WL: Wait list
WOMAC: Western Ontario and McMasters Universities osteoarthritis index
